# S6K2 in Focus: Signaling Pathways, Post-Translational Modifications, and Computational Analysis

**DOI:** 10.3390/ijms26010176

**Published:** 2024-12-28

**Authors:** Mahmoud I. Khalil, Mohamed Helal, Ahmed F. El-Sayed, Rana El Hajj, Jasmine Holail, Marwa Houssein, Ahmed Waraky, Olivier E. Pardo

**Affiliations:** 1 Department of Biological Sciences, Faculty of Sciences, Beirut Arab University, Beirut P.O. Box 11-5020, Lebanon; r.hajj@bau.edu.lb; 2 Molecular Biology Unit, Department of Zoology, Faculty of Science, Alexandria University, Alexandria 21568, Egypt; 3 Department of Biology, University of Southern Denmark, 5230 Odense, Denmark; mhelal@biology.sdu.dk; 4 National Institute of Oceanography and Fisheries (NIOF), Cairo 11516, Egypt; 5 Microbial Genetics Department, Biotechnology Research Institute, National Research Centre, Giza 12622, Egypt; ahmedfikry.nrc@gmail.com; 6 Egypt Center for Research and Regenerative Medicine (ECRRM), Cairo 11517, Egypt; 7 Department of Biochemistry and Molecular Medicine, College of Medicine, Alfaisal University, Riyadh 11533, Saudi Arabia; rb22182@bristol.ac.uk; 8 Translational Health Sciences, Bristol Medical School, University of Bristol, Bristol BS1 3NY, UK; 9 Scientific Support, HVD Life Sciences, Riyadh 11411, Saudi Arabia; housseinmarwa@gmail.com; 10 Region Västra Götaland, Department of Clinical Chemistry, Sahlgrenska University Hospital, 41345 Gothenburg, Sweden; ahmed.waraky@gu.se; 11 Department of Haematology, Cambridge Stem Cell Institute, Cambridge University, Cambridge CB20AW, UK; 12 Department of Laboratory Medicine, University of Gothenburg, 41345 Gothenburg, Sweden; 13 Division of Cancer, Department of Surgery & Cancer, Faculty of Medicine, Imperial College London, London W12 0NN, UK

**Keywords:** S6K2, cell signaling, regulation, post-translational modifications, in silico analysis, cancer, environmental contaminants, molecular dynamics, inhibitors, docking studies

## Abstract

S6 Kinase 2 (S6K2) is a key regulator of cellular signaling and is crucial for cell growth, proliferation, and survival. This review is divided into two parts: the first focuses on the complex network of upstream effectors, downstream modulators, and post-translational modifications (PTMs) that regulate S6K2 activity. We emphasize the dynamic nature of S6K2 regulation, highlighting its critical role in cellular homeostasis and its potential as a therapeutic target in diseases like cancer. The second part utilizes in silico analyses, employing computational tools to model S6K2’s three-dimensional structure and predict its interaction networks. Molecular dynamics simulations and docking studies reveal potential binding sites and interactions with novel known inhibitors. We also examine the effects of environmental contaminants that potentially disrupt S6K2 function and provide insights into the role of external factors that could impact its regulatory mechanisms. These computational findings provide a deeper understanding of the conformational dynamics of S6K2 and its interactions with its inhibitors. Together, this integrated biochemical and computational approach enhances our understanding of S6K2 regulation and identifies potential new therapeutic strategies targeting S6K2 in the oncology setting.

## 1. Introduction

S6K2 (ribosomal protein S6 kinase beta 2), an activated downstream effector of mammalian target of rapamycin (mTOR), is a member of the ribosomal S6 kinase (RSK) family of serine/threonine kinases involved in the regulation of protein synthesis, cell growth, and cell proliferation. S6K2 is reported to play a significant role in malignant cell transformation and proliferation; however, it has been less investigated than its closely related isoform S6K1 [1]. S6K1 and S6K2 demonstrate unique expression patterns across different cell types and conditions. Although both isoforms are present in various tissues, their expression levels differ markedly. The precise ratio of their expression can vary based on the specific cell type, developmental phase, and external stimuli [2,3,4,5].

**Figure 1:**
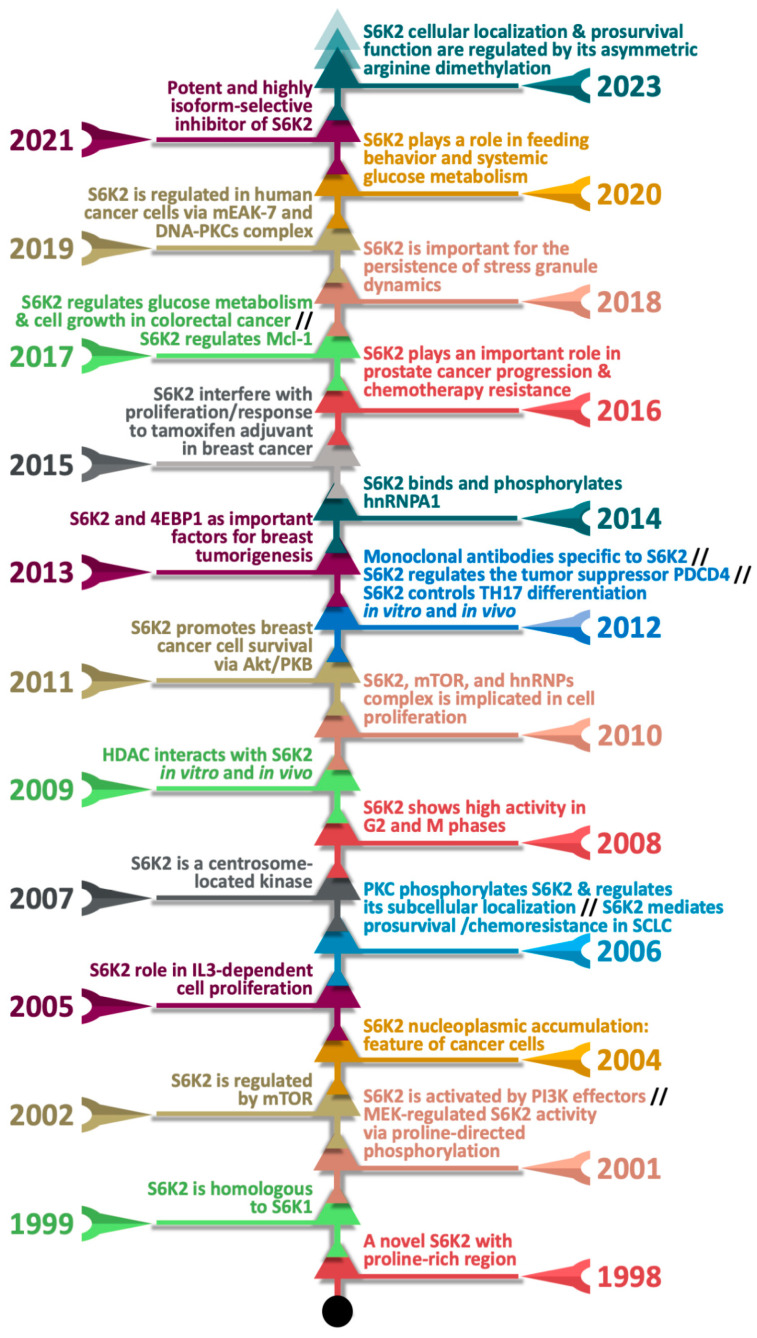
Timeline Highlighting S6K2’s Key Milestones. The figure traces the discovery of S6K2, mechanistic insights into its cellular signaling, its role in cancer biology, and the evolution of therapeutic targeting approaches to inhibit its activity [1,6,7,8,9,10,11,12,13,14,15,16,17,18,19,20,21,22,23,24,25,26,27,28,29,30,31,32,33].

The discovery and characterization of S6K2 can be traced through various studies focused on understanding the molecular mechanisms that control cell growth and metabolism. Figure 1 illustrates a timeline highlighting S6K2’s significant milestones. The aim of this integrative review is, first, to provide a comprehensive overview of S6K2, including its upstream regulators, downstream effectors, and post-translational modifications, and second, to conduct an in silico analysis to identify potential inhibitors of S6K2 and the impact of environmental pollutants, followed by molecular docking for these chemicals.

## 2. S6K2 and Cancer Progression: Signaling Pathways at a Glance

### 2.1. Upstream Cancer-Promoting Regulators of S6K2

#### 2.1.1. mTORC1 Signaling Pathway and S6K2

mTOR is a serine/threonine kinase that orchestrates key processes related to cell growth, survival, proliferation, and protein synthesis in cells [34]. Several oncogenic cellular pathways converge on the mTOR pathway, which has long been recognized as a major player in the activation of the S6K family [2,31,35]. S6K2 is a downstream effector of mTOR, with a well-established role in cancer [36,37,38], albeit to a lesser extent than S6K1. Moreover, studies on S6K2 knockout mice and S6K2 siRNA–treated cells revealed an increased compensatory activity of S6K1, as reflected by a blunt increase in (Thr389) p-S6K1 levels [2]. The examination of the function of Mammalian Enhancer-of-Akt-1-7 (mEAK-7) in mTORC1 signaling reveals that S6K2 promotes cell proliferation and migration in response to mTOR activation in various cancer types, such as hepatocellular carcinoma and lymph node-positive breast cancers [39]. Moreover, the mTORC1/S6K2 pathway is hyper-activated and actively involved in the carcinogenesis of invasive micropapillary breast carcinoma [40]. An intriguing role of S6K2 as a prognostic marker has also been reported in breast cancer. Indeed, a study by Sridharan et al. reported that S6K2, but not S6K1, is involved in the proliferation and survival of breast cancer cells in response to mTOR activation [20,41].

The *S6K2* gene was also found to be amplified in 4.3% of patients with breast cancer [42]. However, despite this low amplification rate, it is correlated with a large number of gains (>3 copies) (21%), attributing the crucial role of S6K2 in breast cancer progression [2,42]. Moreover, *S6K2* overexpression was correlated with increased estrogen receptor (ER^+^) status and Cyclin D1 (CCND1) amplification, as well as progesterone receptor (PgR) expression. This suggests a functional link between S6K2 and ER signaling [42]. Furthermore, the mTORC1/S6K2 signaling pathway is triggered by many mediators, such as increased expression of Ras Homolog Enriched in Brain (RHEB) and decreased activity of AMP-activated Protein Kinase (AMPK) [40]. Regulation of the mTORC1/S6K2 signaling pathway is also modulated by the tuberous sclerosis complex (TSC1/2). Interestingly, proteomic and phospho-proteomic data analyses showed that the inhibitory phosphorylation of TSC1/2 correlates with the activation of mTORC1/S6K2 signaling [40].

In colorectal cancer, a relationship between mTORC1 and S6K2 has been identified, showing a crucial role in promoting cell proliferation, migration, and invasion [43]. In particular, transfection of LoVo colorectal cancer cells with an miR-1273g-3p inhibitor remarkably reduced the mRNA and protein levels of ERBB4, PIK3R3, mTOR, and S6K2 [43]. These results were reversed by inhibiting the cannabinoid receptor using CNR1 siRNA, which promotes cell migration [43].

Furthermore, S6K2 is involved in promoting cell migration and proliferation in prostate cancer [44]. Depletion of S6K2 significantly reduced the viability of the human metastatic prostate cancer cell line PC-3 and has been suggested as a target for restoring docetaxel sensitivity in advanced prostate cancer [27]. These findings highlight the importance of deciphering the molecular mechanisms underlying the mTOR-S6K2 signaling pathways, as these offer valuable insights into potential therapeutic targets for cancer.

The mTOR-S6K pathway is involved in promoting stress granule (SG) assembly in response to cellular stress [45]. Various stress conditions lead to the formation of granules consisting of cytoplasmic mRNA-protein complexes induced by the inhibition of translation initiation. SGs are associated with aging and cancer [45]. Indeed, the induction of acute oxidative stress by the treatment of HeLa cells with 0.5 mM sodium arsenite is mediated by the mTOR-S6K pathway. Robust localization was also observed between the SG markers G3BP1, mTOR, S6K1, and S6K2. Importantly, it is shown that S6K2 knockdown increased the number and the size of SGs. Interestingly, S6K2-mediated activity is dependent on the interaction between mTORC1 and S6K1/S6K2 kinases. S6K1 primarily impacts mTORC1 activity related to cell growth and metabolism. However, S6K2 influences SG formation in response to stress. The increased size and number of SGs due to the knockdown of S6K2 indicate a compensatory mechanism in which reduced S6K2 activity might stabilize SGs, potentially by altering stress response pathways involving mTORC1. Thus, the findings suggest that S6K1 and S6K2 may play different but interconnected roles in cellular stress response [45]. In addition, S6K2 promotes the persistence of SGs, as confirmed by the decrease in the number of cells displaying SGs after late time points of treatment with sodium arsenite in the control and S6K1, but not S6K2-expressing conditions [30]. This has disease relevance as a protective role of stress granules in cancer. Furthermore, the nuclear localization of S6K2 may be mediated by heterogeneous nuclear ribonucleoproteins (hnRNPA1) [30], a protein that relocates to SGs in response to cellular stress. Therefore, investigating the correlation between mTOR-S6K2 upregulation in stress granules and cancer progression is of great interest [30,45].

Dysregulation of mTOR signaling fosters cancer development through a myriad of events, including the involvement of Mammalian Enhancer-of-Akt-1-7 (mEAK-7), which activates alternative mTOR signaling. Multiple studies have revealed increased mRNA levels of human *MEAK7* in tumors, including hepatocellular carcinoma and lymph node-positive breast cancers [46,47]. Knockdown of mEAK-7 resulted in increased cell size, indicating altered S6K1 activity and aberrant cell size regulation. Moreover, overexpression of constitutively active S6K1 or S6K2 rescued the effects of mEAK-7 knockdown, suggesting that mEAK-7 acts upstream of S6K2 and promotes its signaling. These data confirm the regulatory role of mEAK-7 in modulating S6K2-mediated cellular processes through mTOR signaling [39].

The MTOR/S6K pathway also mediates the activity of 4EBP1, the eukaryotic translation initiation factor 4E-binding protein 1, through a pathway that is actively involved in various malignancies. For instance, genome-wide scale analysis of breast tumors reveals a high expression of S6K1, S6K2, and 4EBP1, which are significantly associated with poor prognosis [5]. Moreover, the activity of S6K2 also involves the regulation of the transcription factor E2F, a downstream target of 4EBP1 that plays a crucial role in tumor progression through estrogen-dependent and -independent mechanisms. Activation of E2F is triggered by the RAS/MAPK and PI3K/Akt signaling pathways [48]. Moreover, E2F1 exhibits a feedback loop that suppresses apoptosis by targeting AKT. Silencing S6K2 decreases E2F1 expression levels, and E2F1 activity may also be regulated through the differential localization of S6K2. Indeed, when both S6K2 and 4EBP1 are localized in the nucleus, this correlates with low-grade tumors and better prognosis. These findings confirm the collaborative roles of S6K2 and 4EBP1 in tumor progression by regulating the activity of E2F1 in the cell cycle [5,24,49].

#### 2.1.2. Regulation of S6K2 Activity by Phosphoinositide-Dependent Kinase-1 (PDK1)

PDK1 is an AGC kinase that acts as an upstream mediator of S6Ks through the mTORC1 signaling pathway. In this context, the activation of S6K2 is mediated by the activation of PDK1, albeit to a lesser extent than that of S6K1. Indeed, while PDK1 is involved in the activation of both S6K1 and S6K2, PDK1 exhibits a stronger interaction with S6K1, while its effect on S6K2 is less pronounced. This differential effect is primarily due to the structural and functional differences between the two proteins. In particular, S6K1 is predominantly a cytoplasmic isoform involved in the regulation of protein synthesis, tightly aligning with the role of PDK1 in mediating growth signals. In contrast, frequent localization of S6K2 in the nucleus involves other regulatory pathways that are not strictly dependent on PDK1. S6K2 is phosphorylated at three serine residues on its C-terminus, S410, S417, and S423, downstream of the MEK/ERK signaling pathway. The phosphorylation of these sites constitutes the initiation step in the activation process by exposing the internal region of S6K2. The latter is then phosphorylated by PDK1 at T288, leading to full activation of the kinase [50,51]. In addition, the activation of S6K2 occurs in a sequential manner introduced by the phosphorylation of the three proline-directed serine residues in the autoinhibitory domain, Ser-410, Ser-417, and Ser-423, downstream of MEK/ERK signaling. Afterward, phosphorylation of Ser-370 then permits phosphorylation of Thr-288 by the mTORC1 complex, followed by that of Thr-228 by PDK1 [1,15].

#### 2.1.3. Regulation of S6K2 Signaling by Akt

The Akt pathway is extensively involved in various cellular processes, such as the control of cell growth, proliferation, and survival [52]. The knockdown of S6K2 decreases Akt phosphorylation and subsequent cell survival in MCF-7 cells [20]. Interestingly, the expression of constitutively active Akt (CA-Akt) in MCF-7 cells reduced TNF-induced poly-ADP ribose polymerase (PARP) cleavage [20], while S6K2 knockdown significantly increased TNF-induced PARP cleavage. However, the overexpression of CA-Akt repressed TNF-induced PARP cleavage in S6K2-depleted cells. These findings strongly confirm the pro-survival role of S6K2 in cancer cells through the Akt pathway [20]. Further experiments revealed that silencing S6K2 increased TNF-induced p53 levels, whereas silencing p53 decreased Bid levels, a pro-apoptotic protein belonging to the Bcl-2 family. Thus, S6K2, activated by AKT, downregulates Bid through inhibition of p53 [20,53]. Taken together, these facts suggest that activation of S6K2 by Akt plays a pivotal role in S6K2’s impact on cancer therapy [20].

#### 2.1.4. Fibroblast Growth Factor (FGF2)-S6K2 Signaling Pathway

Fibroblast Growth Factor 2 (FGF2) is involved in mediating resistance to cell death [15,54,55]. The activation of FGF2 signaling leads to the assembly of a protein complex comprising B-Raf, protein kinase C-epsilon, and S6K2. This complex is involved in mediating chemoresistance in small-cell lung cancer and promoting the translation of molecules linked to tumorigenesis [15,22,25]. Silencing of S6K2 reduces the viability of small-cell lung cancer cells and non-small-cell lung cancer cells by preventing the formation of this complex [1,15].

#### 2.1.5. Inhibition of S6K2 Activity by Histone Deacetylase (HDAC)

Histone acetylation plays a role in S6K2 activity. Indeed, acetylation stabilizes S6K2 and its activity, leading to its survival and chemoresistance in HEK293 cells [18]. HDAC inhibition increased the protein levels of S6Ks, and S6K2 was stabilized upon treatment of cells with HDAC inhibitors [18,56]. Moreover, S6K2 seems completely reliant on HDAC, exhibiting differential regulation among S6K isoforms. The involvement of HDACs may provide a further link between these kinases and their transcriptional machinery. The acetylation-mediated stabilization of S6K2 coupled with the pro-survival and drug-resistant phenotypes related to the overexpression of this kinase may negatively impact the clinical efficiency of HDAC inhibitors.

#### 2.1.6. Regulation of S6K2 by Protein Arginine Methyltransferases (PRMTs)

The localization of S6K2 plays a crucial role in chemoresistance and survival of various cancer types [12]. Khalil et al. revealed that post-translational modifications of S6K2 promote its nuclear localization and protect it against starvation-induced cell death [33]. S6K2 harbors dual bi-arginine (RXR) motives within its nuclear localization signal (NLS), which are targeted by PRMTs [57,58]. Co-immunoprecipitation assays in HEK293 cells revealed that S6K2 interacts with PRMT1, 3, and 6. Furthermore, S6K2 was exclusively shown to be methylated by PRMT1, 3, and 6 in vitro and in vivo. Indeed, methylation of S6K2 occurred at Arginines 475 and 477, suggesting that methylation modulates S6K2 DNA-binding affinity and function by inducing its nuclear localization. Indeed, the pro-survival role of methylated S6K2 was highlighted by the increased cell death observed in cells expressing the methylation-dead S6K2 mutant. Finally, PP2A, known as the chief phosphatase of the S6K family, binds PRMT1 and inhibits its methyltransferase activity [59]. Thus, activation of PP2A leads to the inactivation of S6K2, favors its exclusion from the nucleus, and interferes with its pro-survival activity [33].

### 2.2. Downstream Effectors of S6K2 Promoting Cancer Progression

#### 2.2.1. Regulation of the Activity of Ribosomal S6 Protein by S6K2

Some studies have suggested that there is no apparent association between the phosphorylation of S6 protein and the expression levels of either S6K1 or S6K2 in endometrial and breast cancers [3,12]. These findings are contradictory to other studies reporting that, in contrast to S6K1, S6K2 is involved in regulating the activity of downstream S6 ribosomal proteins [37,40,60]. Indeed, S6K2 knockout mice, more so than S6K1 mice, exhibited a sharp decrease in the cellular levels of S6 phosphorylation, although both S6K1 and S6K2 are essential for complete phosphorylation of S6 [60]. Furthermore, the activity of S6K2 in S6 phosphorylation is mediated by the mitogen-activated protein kinase (MEK) pathway. However, in vitro treatment with the MEK inhibitor AZD6244 also shows an additive reduction in the phosphorylation of S6 in cells with silenced S6K1 and S6K2 expression [61,62]. Moreover, deficiency of S6K2 notably reduces the phosphorylation of S6 ribosomal protein in lung homogenates and isolated mesenchymal cells following TGF-α expression [63]. These findings suggest that targeting S6K2, whether alone or in combination with S6K1, could be a prospective therapeutic approach for direct S6 inhibition in various cancer types to limit cell proliferation and migration [62].

#### 2.2.2. Regulation of Histone H3 Phosphorylation by S6K2 and Associated Cellular Functions

S6K2 phosphorylates Histone H3 to control cell proliferation and differentiation [38]. Indeed, S6K2 phosphorylates core histones H2A, H2B, and H3 in vitro. Interestingly, investigating the phosphorylation of these histones incorporated into the nucleosome revealed that only H3 was phosphorylated at the Thr45 residue in HEK293T cells [38]. Increased levels of Thr45-phosphorylated H3 were obtained during the differentiation of the leukemic cell lines THP-1 and HL-60 [64], which may suggest the involvement of S6K2 in this process. Moreover, the phosphorylation of H3 was positively correlated with mTOR pathway-based regulation of S6Ks activity, as shown by experiments using the mTOR inhibitor rapamycin. These findings highlight the biological importance of histone H3-Thr45 phosphorylation downstream of the mTOR/S6K2 axis.

#### 2.2.3. S6K2-Mediated Phosphorylation of Heterogeneous Nuclear Ribonucleoprotein A1 (hnRNPA1)

S6K2 mediates the pro-survival activity of FGF2 by triggering the translation of the anti-apoptotic proteins B-cell lymphoma-extra-large (BcL-XL) and X-chromosome-linked inhibitors of apoptosis protein (XIAP) in different cell types, including H-510 SCLC and HEK-293 cells. It has been reported that the RNA-binding proteins programmed cell death 4 (PDCD4) [4] and hnRNPA1 interact with S6K2 following FGF2 stimulation. A strong association between S6K2 and hnRNPA1 was confirmed by co-immunoprecipitation in both H510 SCLC and HEK-293 cells [25]. Furthermore, an increase in S6K2 activity was associated with the phosphorylation of hnRNPA1 on Ser4/6, which promoted the binding of this protein to, and subsequent nuclear export of, the mRNAs for BcL-XL and XIAP through binding to their 5’-UTR. This leads to their increased translation and induction of resistance to cell death triggered by several cytotoxic drugs in lung, breast, osteosarcoma, and prostate cancer cells [15,25,65]. Indeed, the dissociation of phosphorylated hnRNPA1 from its cargo mRNAs occurs in the cytoplasm, leading to elevated IRES-mediated translation. Hence, in breast and lung cancer patient samples, increased expression of S6K2 was associated with reduced cytoplasmic hnRNPA1 and increased levels of BcL-XL. This process plays a pivotal role in the pro-survival effects of FGF-2/S6K2 signaling. Elsewhere, it was found that the S6K2-mediated phosphorylation of hnRNPA1 on Ser6 controls glucose metabolic reprogramming, highlighting the role of S6K2 as a potential therapeutic target [25].

#### 2.2.4. Phosphorylation of PDCD4 and Upregulation of Anti-Apoptotic Proteins by S6K2

The family of Bcl2 proteins is significantly regulated by S6K2 [20,25]. Regulation of PDCD4 by S6K2 has been extensively studied. Indeed, the knockdown of S6K2 enhanced the sensitivity of prostate cancer cells to docetaxel. In this context, S6K2 is activated by FGF-2 and phosphorylates PDCD4 [25]. Upon phosphorylation, PDCD4 is degraded, allowing the translation of the anti-apoptotic proteins XIAP and BcL-XL, thus promoting chemoresistance and survival of lung cancer cells [15,22,27]. Other reports have shed light on the role of S6K2 in the modulation of Mcl-1 and BcL-XL activities through the phosphorylation of PDCD4. Knockdown of S6K2 decreased BcL-XL translation levels. Depletion of S6K2 also enhances apoptosis by decreasing Mcl-1 levels in T47D cells [29]. Depletion of PDCD4 reverses BcL-XL downregulation yet fails to rescue Mcl-1 levels or counteract the effect of S6K2 knockdown on doxorubicin-induced apoptosis. While alteration of Akt activity did not significantly impact Mcl-1 downregulation by S6K2 deficiency, increased JNK levels were obtained following S6K2 silencing, and knockdown of JNK1 partially restored Mcl-1 levels. Furthermore, the downregulation of Mcl-1 by S6K2 deficiency was partially rescued by the proteasome inhibitor MG132, suggesting a role of S6K2 in regulating the proteasomal degradation of Mcl-1.

#### 2.2.5. S6K2 Controls the Transactivation Response RNA-Binding Protein (TRBP) Through mTOR Pathway

S6K2 is activated downstream of mTOR and mediates the TRBP signaling pathway by augmenting miRNA biogenesis. In primary human dermal lymphatic endothelial cells (HDLEC), S6K2 displays a differential effect than S6K1 in the mediation of cell proliferation through TRBP activation [15,66]. In addition, Warner et al. demonstrated that TRBP expression and phosphorylation levels were impaired in S6K2-deficient HDLECs. The interaction between S6K2 and TRBP was significant only 30 min after Angiopoietin-1 (ANG1) addition, while the interaction between S6K1 and TRBP was comparable to that of the negative control cells, thus suggesting a specific link between TRBP and S6K2 [66]. Furthermore, the interaction between S6K2 and TRBP increased miRNA expression, as the depletion of S6K2 lowers the expression of ANG1-induced miRNAs. Conversely, the depletion of S6K1 augments miRNA expression, and the concurrent depletion of both S6K1 and S6K2 further decreases miRNA levels. These findings highlight the complex role of S6Ks in TRBP-mediated miRNA biogenesis, particularly S6K2-mediated regulation of TRBP as an influential factor in human primary lymphatic endothelial cells [66].

#### 2.2.6. Regulation of the Transcription Factor YY1 by S6K2

**Figure 2:**
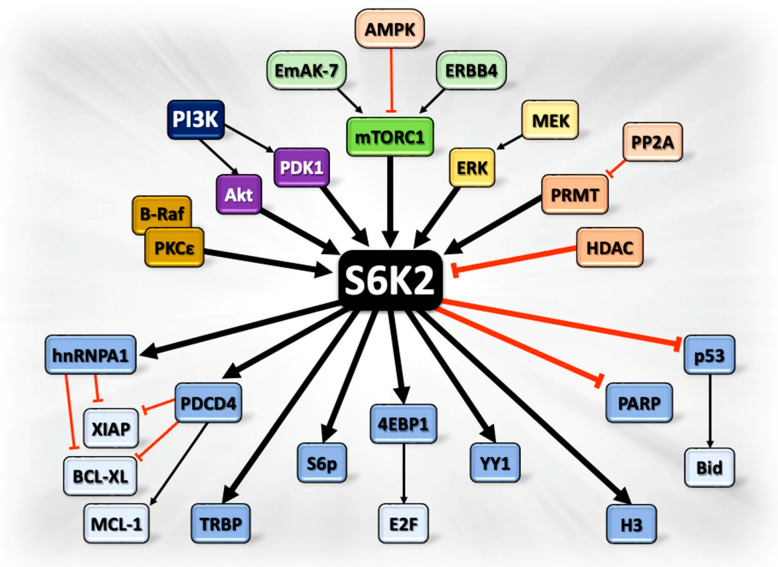
Major Upstream regulators and downstream effectors of S6K2. The figure shows the major proteins involved in the activation, localization, expression, and functions of S6K2.

The altered expression of the Yin Yang (YY1) transcription factor has been reported in various tumors due to its involvement in many cellular processes, such as cell proliferation and apoptosis. S6K2 mediates the expression of YY1 through the formation of a complex in a panel of mammalian cell lines [67]. The latter study was the first to show that YY1 is a transcription factor that binds specifically to S6K2. Co-immunoprecipitation assays in several cancer cell lines confirmed this interaction, notably facilitated by the C-terminal regulatory domain of S6K2 after stimulation with a mitogen [56,67,68]. The S6K2/YY1 interaction is rapamycin-sensitive, with rapamycin inhibiting the formation of the serum-induced S6K2/YY1 complex. These findings demonstrate the involvement of mTOR in this signaling event. However, the physiological function of this interaction remains to be understood [56,67]. Figure 2 illustrates the major upstream and downstream effectors of S6K2, as discussed above.

## 3. Major Post-Translational Modifications of S6K2

S6K2 plays a crucial role in regulating ribosome biogenesis and translation [68,69]. S6K2 activity is influenced by post-translational modifications, particularly phosphorylation [8]. In addition to phosphorylation, S6K2 is also a target of arginine methylation [33], ubiquitination [70,71], and acetylation of a lysine residue close to the C-terminal domain [18]. The activation of S6K2 in response to different growth factors, cytokines, nutrients, and cellular stresses leads to the phosphorylation of numerous targets related to protein synthesis and RNA metabolism. This process enhances the translational ability of the cell, ultimately promoting cell growth [8].

**Figure 3:**
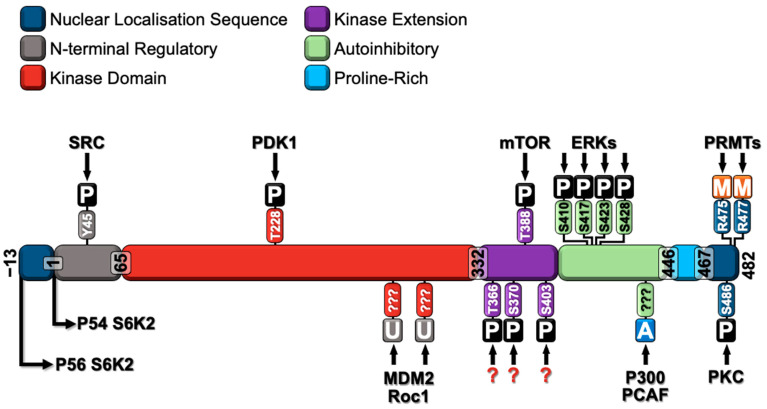
Protein domain organization of S6K2 showing the major post-translational modification sites of S6K2 and the proteins involved in these modifications. The long isoform of S6K2 (P56 S6K2) possesses an additional nuclear localization sequence that is absent in the short isoform of S6K2 (P54 S6K2). The amino acids involved in each post-translational modification are annotated with their positions. P, phosphorylation; U, ubiquitination; A, acetylation; M, methylation; Y, tyrosine; T, threonine; S, serine; R, arginine; ??? and an unidentified amino acid.

The kinase domain of S6K2 exhibits 83% amino acid identity with that of S6K1 [68]. The principal sequence divergence between S6K1 and S6K2 occurs at their C- and N-termini. Both S6K1 and S6K2 possess several essential residues that play a role in kinase activation [56]. Of the eight identified serine/threonine phosphorylation sites on S6K1, seven (Thr-228, Ser-370, Thr-388, Ser-403, Ser-410, Ser-417, and Ser-423 on p54S6K2) are conserved in S6K2 [56]. Additionally, both isoforms are subject to acetylation and ubiquitination [18,71]. However, only S6K2 is modified by methylation, which is attributable to the presence of two overlapping arginine-rich (RXR) motifs in its C-terminal domain that are absent in S6K1 [33]. Figure 3 illustrates the major post-translational modifications of S6K2, as discussed below.

### 3.1. S6K2 Phosphorylation

Phosphorylation serves as a common cellular post-translational modification that regulates protein function [69]. In the context of S6K2, phosphorylation at distinct sites impacts both its activity and its capacity to phosphorylate its major substrate, ribosomal protein S6. S6K1 and S6K2 share several crucial residues that are involved in kinase activation. The mitogen-activated protein kinase/extracellular signal-regulated kinases (MAPK/ERKs) and PI3K/mTOR pathways are both recognized for their role in S6K2 phosphorylation [11]. The activation of S6K2 involves a stepwise process [72]. Initial phosphorylation of the S6K2 autoinhibitory domain, particularly at Ser-410, Ser-417, and Ser-423 downstream of MAPK/ERK signaling, is crucial for overcoming the suppression imposed by the C-terminal autoinhibitory pseudo-substrate domain [73]. The MAPK/ERK-dependent pathway involves activation of MEK, which in turn phosphorylates and activates ERKs [74]. Activated ERKs can directly phosphorylate and activate S6K2, leading to enhanced ribosome biogenesis and translation [72]. This pathway is typically activated in response to growth factors and mitogens, and it serves as a link between extracellular signals and cellular processes involved in protein synthesis [72]. This is followed by the phosphorylation of Ser-370, which then allows for the phosphorylation of Thr-388 by the mTORC1 complex, and finally, Thr-228 by 3-phosphoinositide-dependent kinase 1 (PDK1). The PI3K/mTOR pathway is another major regulator of S6K [11], with mTORC1 playing a central role by acting as a sensor of nutrient availability and cellular energy status [75]. Activation of mTOR leads to the phosphorylation and activation of S6K. Phosphoinositide 3-kinase (PI3K) is an upstream mediator of the mTOR pathway, which is activated by various growth factors. Through the generation of phosphatidylinositol triphosphate (PIP3), it activates downstream signaling events that converge on mTORC1 [75]. Hence, the PI3K/mTOR pathway integrates cues from growth factor signaling, nutrient availability, and cellular energy levels to control S6K activation and protein synthesis [2].

It is worth highlighting that the distinct regulation of S6K1 and S6K2 in relation to nutrient deprivation and mTOR inhibition indicates that these isoforms play different roles and operate through separate signaling pathways within the mTOR signaling network [50,56]. Conducting additional research to understand the mechanisms underlying their differential regulation can offer valuable insights into their functions and potential therapeutic applications. S6K2, unlike S6K1, can be phosphorylated and activated by protein kinase C (PKC) both in vitro and in vivo [14]. The site of PKC phosphorylation on S6K2 was identified as Ser-486 [14]. Interestingly, while this phosphorylation does not affect the intrinsic enzymatic activity of S6K2, it impairs the function of the nuclear localization signal (NLS) within S6K2 [14]. As a result, phosphorylation of S486 by PKC leads to the cytoplasmic accumulation of S6K2 upon cell stimulation with PKC agonists like phorbol 12-myristate 13-acetate (PMA) [14].

In addition to serine/threonine phosphorylation, both S6K1 and S6K2 can undergo tyrosine phosphorylation downstream of receptor tyrosine kinase (RTK) activation [56]. Both S6K isoforms are associated with several RTKs, including the platelet-derived growth factor receptor (PDGFR), hepatocyte growth factor receptor (HGFR), and colony-stimulating factor receptor (CSFR). Upon activation of these receptors, N-terminal tyrosine phosphorylation of S6Ks was observed specifically at Tyr-39 on S6K1 and Tyr-45 on S6K2. These tyrosine phosphorylation events were found to occur in an Src family kinase (SFKs)-dependent manner [75]. Interestingly, this tyrosine phosphorylation does not result in modulation of the catalytic activity of S6Ks or their gross subcellular redistribution. While this tyrosine phosphorylation appears to be shared between S6K1 and S6K2 in vitro, only S6K2 was found to be phosphorylated in response to Fyn transgene expression in vivo [56,75]. This suggests that the two S6K isoforms may be differentially wired to SRC family members through distinct cellular multi-protein complexes, leading to isoform-specific tyrosine phosphorylation patterns in response to RTK activation [56].

### 3.2. S6K2 Methylation

The C-terminus of S6K2, unlike that of S6K1, possesses two arginine-rich (RXR) motifs that overlap [33]. These RXR motifs undergo post-translational modification via arginine methylation, a process catalyzed by protein arginine methyltransferase (PRMT) enzymes [57]. The arginine methylation of RXR motifs at the C-terminus of S6K2 facilitates its localization within the nucleus [33]. The nuclear localization of S6K2 is thought to play a significant role in cellular survival mechanisms [33,57]. These discoveries indicate that S6K2 nuclear localization may contribute to the progression of small-cell lung cancer (SCLC) cells and the development of chemoresistance where arginine methylation is elevated [33].

### 3.3. S6K2 Acetylation

Lysine acetylation plays a crucial role in modifying the function, localization, protein stability, and interactions of S6Ks [18]. Upon mitogen stimulation, S6Ks interact with p300 and p300/CBP-associated factor (PCAF) acetyltransferases [18]. In vitro, p300 and PCAF can acetylate S6Ks, and the acetylation of S6Ks has been observed in cells expressing p300. The acetylation sites targeted by p300 are located within the divergent C-terminal regulatory domains of both S6K1 and S6K2. Acetylation of these sites is enhanced when class I/II histone deacetylases (HDACs) are inhibited by trichostatin-A [56]. Moreover, the increase in S6K1 acetylation by nicotinamide implies that sirtuin deacetylases play a role in the deacetylation of S6K. Sirt proteins belong to the Sir2 family of NAD^+^-dependent protein deacetylases in mammals [76,77]. Both the expression of p300 and inhibition of HDACs lead to increased S6K protein levels, with S6K2 being stabilized in cells treated with HDAC inhibitors [18]. The discovery that S6Ks are targeted by histone acetyltransferases revealed a new level of crosstalk between mitogenic signaling pathways and transcriptional machinery, adding complexity to the regulation of S6K function [18]

### 3.4. S6K2 Ubiquitination

It has been shown that the steady-state levels of S6K1 and S6K2 are controlled by ubiquitination followed by proteasomal degradation [70]. The ubiquitination of S6Ks is coordinated by signaling pathways induced by mitogenic stimuli and extracellular stress, independent of S6K phosphorylation and subsequent activation [70,71]. However, the plethora of functions of this modification is still unclear and needs further investigation [71].

## 4. Inhibition of S6K2 in Cancer

The remarkable therapeutic success achieved with rapamycin and its analogs underscores the potential effectiveness of targeting the p70S6K pathway for cancer treatment [78]. Earlier studies in knockout mice have shown that both S6K1 and S6K2 are required for full phosphorylation of S6, but that S6K2 may be the major effector for this phosphorylation [60]. The pan-p70S6K inhibitor LY2584702 has shown promising results both in vitro and in vivo [79,80]. However, despite these promising preclinical results, its efficacy did not translate into the clinical setting [79]. Patients treated with LY2584702 alone [79] or in combination with everolimus [79] did not show significant partial or complete responses, and higher doses of LY2584702 were not tolerated by patients [5]. This is consistent with several lines of evidence suggesting that S6K2 targeting should be selective without affecting S6K1 activity [56]. This is highlighted by the observation that unlike single S6K2 knockout mice, mice lacking both S6K1 and S6K2 experienced perinatal lethality [60], which implies the requirement for at least one isoform for normal homeostasis. Based on the current literature, only two inhibitors with significant selectivity for S6K1 over S6K2 in biochemical assays have been identified to date: FL772 [62] and PF-4708671 [81], and only one S6K2 inhibitor with selectivity over S6K1 [1]. The S6K1 inhibitor FL772 inhibits S6 phosphorylation only in yeast, but not in human cells [62]. In contrast, PF-4708671 has demonstrated efficacy by inducing cell death in tamoxifen-resistant MCF-7 cells through the reduction of the anti-apoptotic protein Mcl-1 and survival [82,83]. Additionally, PF-4708671 exhibited inhibitory effects on cell proliferation and invasion in NSCLC cell lines (A549, SK-MES-1, and NCI-H460), leading to cell cycle arrest in the G0-G1 phase, with promising results observed in vivo as well [84]. To our knowledge, there are no published findings regarding the in vitro or in vivo effects of a recently identified selective S6K2 inhibitor. However, previous studies on the selective silencing of S6K2, but not S6K1, in the breast cancer cell line T47D have shown reduced levels of Mcl-1 and BcL-XL, enhancing apoptosis induced by TRAIL and doxorubicin [29]. It also selectively induces cell death in NRAS-mutant melanoma cells that are resistant to MAPK inhibition [85]. Although the literature identifies only two inhibitors with significant selectivity for S6K1 over S6K2, we used the PubChem database to search for other possible S6K1 and S6K2 inhibitors. This search included data from patent offices, chemical vendors, pharmaceutical companies, public databases, and repositories from other publications that may indirectly contain information on S6K2 inhibitors from large datasets of inhibitors. This revealed 5448 compounds for S6K1, of which 2592 demonstrated activity. For S6K2, 306 compounds were identified, of which 34 showed activity (Supplementary Table S1). Among the active compounds for S6K2, 12 exhibited activity exclusively against S6K2 and not S6K1 (Table 1). Further prediction analysis of these 12 compounds targeting S6K2 using the SwissTarget Prediction engine revealed that the majority were high-probability hits (see Table 1).

**Figure 4:**
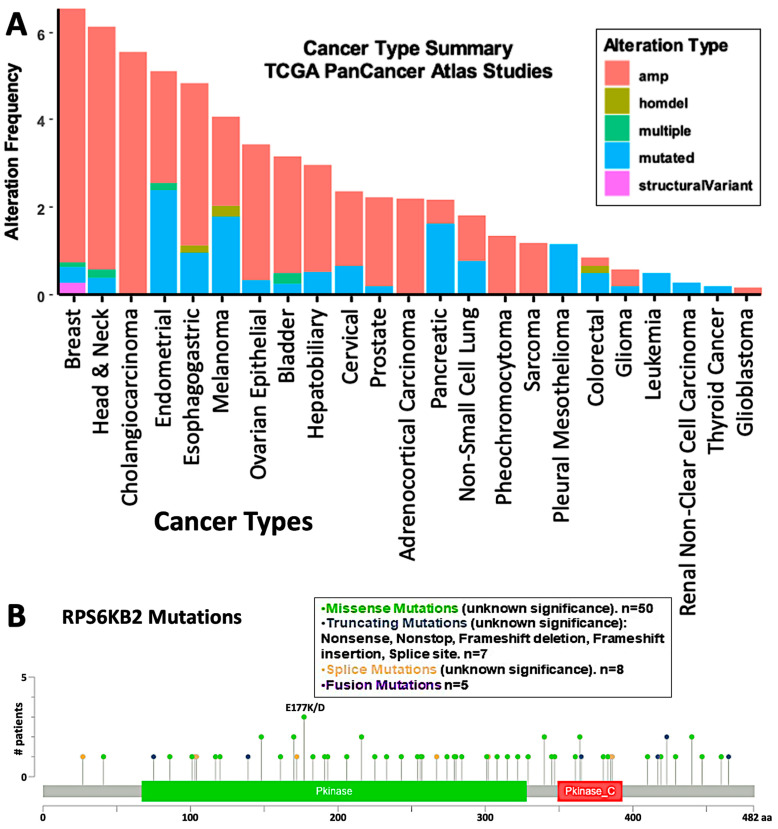
Alteration frequency of RPS6KB2 in TCGA pan-cancer atlas studies. (**A**) Histogram showing the alteration frequency of the RPS6KB2 gene in different cancers based on TCGA pan-cancer analyses (www.cbioportal.org/study/summary?id=5c8a7d55e4b046111fee2296; accessed on 2 December 2024). amp: Amplification, homdel: Homozygous Deletion, multiple: Multiple alterations. (**B**) Analysis of the mutation status of RPS6KB2 in different cancers retrieved from cBioPortal (https://www.cbioportal.org/results/mutations?tab_index=tab_visualize&Action=Submit&session_id=67518aaa83e9543d619338c2&plots_horz_selection=%7B%7D&plots_vert_selection=%7B%7D&plots_coloring_selection=%7B%7D; accessed on 2 December 2024).

**Figure 5:**
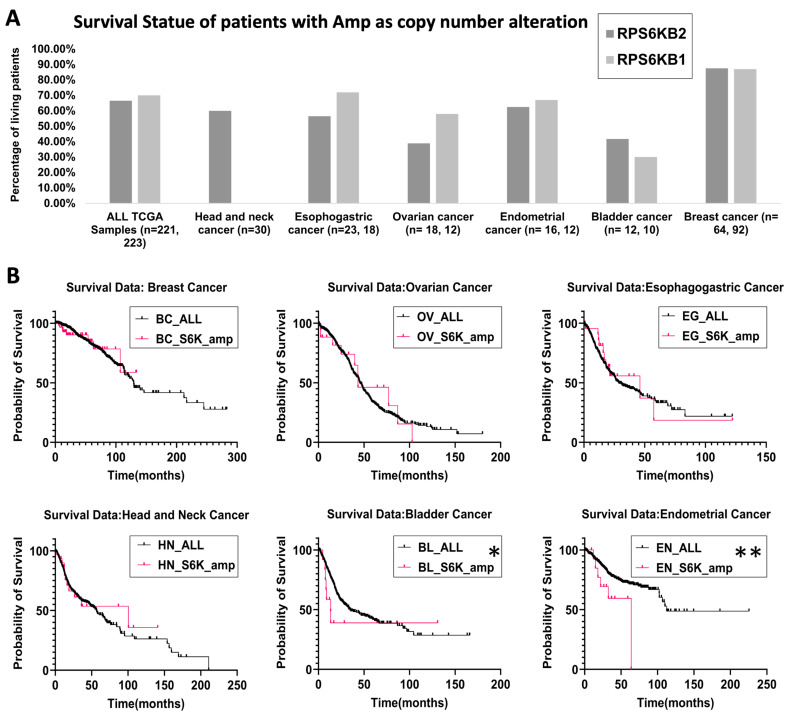
Survival analysis of patients with *RPS6KB2* amplification from the TCGA pan-cancer atlas. (**A**) Histogram showing the overall survival status of patients with amplification of *RPS6KB2* and *RPS6KB1* genes. (**B**) Kaplan–Meier survival curves comparing the overall survival of patients with *RPS6KB2* amplification (red line) versus patients without amplification (black line). BC: Breast Cancer, OV: Ovarian Cancer, EG: Esophagogastric Cancer, HN: Head and Neck cancer, BL: Bladder cancer, EN: Endometrial cancer. Results are significant at *p*-value ≤ 0.05 using the Gehan–Breslaw–Wilcoxon test (*) or using the Log-rank (Mantel–Cox) test (**). Data was retrieved from the cBioPortal interface of the TCGA database.

Next, to investigate the relevance of targeting S6K2 in different types of cancer, we checked the expression levels and the copy number alterations of *RPS6KB2*, the gene encoding S6K2, using the GEPIA and cBioPortal interfaces on the TCGA and GTEx databases. The cBioPortal interface was used to analyze the alteration frequency of the *RPS6KB2* gene in different cancers based on the TCGA pan-cancer dataset. As shown in Figure 4A, breast cancer, head and neck cancer, cholangiocarcinoma, endometrial cancer, esophagogastric cancer, and ovarian and bladder cancer exhibited the highest alteration frequency, with amplification of *RPS6KB2* being the most common copy number alteration. Mutations in *RPS6KB2* were mainly missense mutations of unknown significance (Figure 4B). Amplification of *RPS6KB2* was associated with an overall survival rate of ≤70% among patients with all previously mentioned cancer types, except for breast cancer, where patients with *RPS6KB2* amplification had an overall survival rate of 86.5%. (Figure 5A and Supplementary Table S2). Further analysis of the survival rate of patients with *RPS6KB2* amplification versus those without in the above-mentioned cancer subtypes revealed a significant decline in the survival rate for patients with *RPS6KB2* amplification in bladder and endometrial cancers (Figure 5B and Supplementary Table S3).

A comparison of alteration frequencies between RPS6KB2 and RPS6KB1 in the TCGA pan-cancer dataset showed similar patterns, with amplification being the most common alteration across cancers, such as breast, endometrial, esophagogastric, ovarian, and bladder cancers. However, head and neck cancers and cholangiocarcinoma displayed a higher frequency of RPS6KB2 alterations compared to RPS6KB1 (Supplementary Table S4). Interestingly, while RPS6KB1 amplification had comparable survival impacts in the previously mentioned cancers, RPS6KB2 amplification was associated with lower survival rates in esophagogastric and ovarian cancers (Figure 5A).

**Figure 6:**
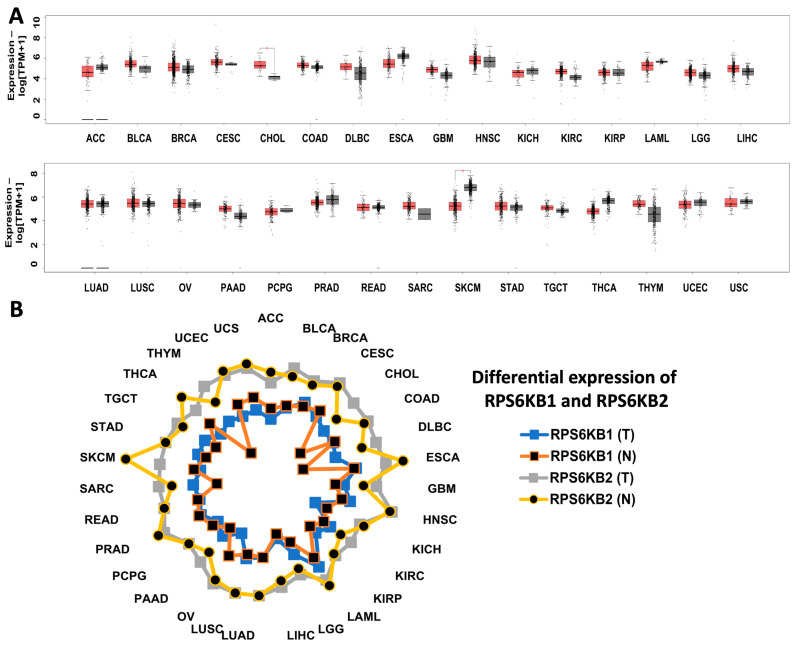
The differential expression of RPS6KB1 and RPS6KB2 genes within the GEPIA database between tumor and normal tissues. (**A**) Boxplots showing RPS6KB2 expression as Log[TPM+1] in tumor tissues (T, red box) and normal tissues (N, gray box). The asterisk (*) indicates a statistical difference in comparison with normal tissues (*p* < 0.01), with Log2FC of 1 as the cutoff value. Tumor-type abbreviations are available at: https://gdc.cancer.gov/resources-tcga-users/tcga-code-tables/tcga-study-abbreviations, (accessed on 2 December 2024). The following numbers per tumor and normal tissues were used accordingly; T = 88; N = 128, T = 404; N = 28, T = 1085; N = 291, T = 306; N = 13, T = 36; N = 9, T = 275; N = 349, T = 47; N = 337, T = 182; N = 286, T = 163; N = 207, T = 519; N = 44, T = 66; N = 53, T = 523; N = 100, T = 286; N = 60, T = 173; N = 70, T = 518; N = 207, T = 369; N = 160, T = 483; N = 347, T = 486; N = 338, T = 426; N = 88, T = 179; N = 171, T = 182; N = 3, T = 492; N = 152, T = 92; N = 318, T = 262; N = 2, T = 461; N = 598, T = 408; N = 211, T = 137; N = 165, T = 512; N = 337, T = 118; N = 339, T = 174; N = 91, T = 57; N = 78. (**B**) Radar chart showing the differential expression of RPS6KB1 and RPS6KB2 between tumor tissues (T) and normal tissues (N) as Log[TPM+1].

The mRNA expression of *RPS6KB2* across cancer subtypes generally mirrored its expression in normal tissues, except for cholangiocarcinoma, where significantly higher tumor-specific expression is evident (Figure 6A). This contrasts with *RPS6KB1*, which shows elevated expression in cholangiocarcinoma, diffuse large B-cell lymphoma (DLBCL), and thymoma (THYM) (Figure 6B). Importantly, *RPS6KB2* consistently demonstrated higher expression levels than *RPS6KB1* in both normal and cancerous tissues, which may suggest a more prominent role for S6K2 in certain cellular processes. This pronounced expression of *RPS6KB2* in cholangiocarcinoma may be attributed to differences in transcriptional regulation, unique subcellular localization, or interactions with specific protein complexes, suggesting its distinct oncogenic functions in this cancer subtype. Taken together, these data suggest the relevance of targeting S6K2 in cholangiocarcinoma, bladder, endometrial, esophagogastric, ovarian, head, and neck, and to a lesser extent in breast cancer. This was confirmed by several recent studies highlighting the correlation between mTOR activation and the development of tumors and drug resistance in biliary tract cancer, with encouraging outcomes observed with the use of Rapalogs in advanced cases [86]. Additionally, mTOR inhibitors have demonstrated favorable outcomes in combination therapies for head and neck cancer [87], as monotherapy in esophagogastric cancer [88], and in estrogen receptor-positive (ER+) breast cancer patients after aromatase inhibitor (AI) failure [89].

## 5. Impact of Environmental Contaminants on Ribosomal Protein Kinase Activity

Oncogenic cellular transformation is dependent on either intrinsic cellular alterations or external oncogenic stimuli. Among external ones is exposure to several environmental contaminants that can trigger cellular transformation, oncogenic initiation, and progression [90,91]. The association between exposure to certain environmental pollutants and S6K2 phosphorylation in the context of cellular activity and oncogenic transformation is lacking; therefore, this section will highlight the impact of different contaminants and ribosomal protein kinase activation and phosphorylation in general.

Polycyclic aromatic hydrocarbon (PAH) exposure can impact and stimulate S6K1 activity. The transcriptomic response of adult male transgenic mouse (Muta^TM^Mouse) exposed to various concentrations (25–100 mg/kg-bw/day) of benzo(b)fluoranthene (BbF) via inhalation for 28 days affected *s6k1* gene activity by modulating the inhibitor of P70 S6 Kinase (P70S6KI) signaling pathway [92]. In contrast, exposure of rat esophageal epithelial cells (RE-149) to Benzo[a] pyrene (B[a]P), another PAH carcinogen found in tobacco and the environment, at concentrations ranging from 0.25 to 2 µM for 48 h induced cellular carcinogenesis via nitric oxide synthase induction (iNOS), which was independent of the mTOR/p70S6K pathway [93].

Similarly, exposure of different types of cells to heavy metals affects several cellular pathways via phosphorylation and activation of S6K1 and 2. Experiments have suggested that S6K activity is dependent on magnesium ions and that this could be blocked by low concentrations of manganese [94]. Treatment of Swiss 3T3 cells with zinc sulfate for four hours at doses of 50–100 µM induced s6k1 activation and phosphorylation in a dose-dependent manner as early as 30 min after treatment. Moreover, treating cells with the mTOR inhibitor rapamycin or the PI3K inhibitors wortmannin and LY294002 led to the inhibition of zinc-induced activation of S6k1. This activation was independent of the PKC pathway and acted upstream of the mTOR/FRAP/RAFT and PI3K pathways. These findings demonstrate that Zn activates S6k1 through PI3K-dependent signaling pathways [95]. S6k1 is also implicated in heavy metal-induced nephrotoxicity [96]. The AKT/mTOR pathway is among the main regulators of cellular autophagic flux [97]. Rat kidney proximal tubule (rPT) cells treated with lead nitrate for 12 h at 0.5 µM showed impaired autophagy flux via dysregulation of the AMPK/mTOR pathway that was associated with inhibition of S6k1 phosphorylation [98]. Similarly, exposure of rat kidney cortex and HK2 cells to potassium dichromate also inhibited autophagy flux via mTOR phosphorylation and S6K1 phosphorylation [99]. In addition, the potent induction of apoptosis by arsenic trioxide (As_2_O_3_) in leukemia cells, which involves AKT/mTOR phosphorylation, is lost upon double-targeted disruption of S6Ks (S6K1^−/−^/S6K2^−/−^), indicating that apoptosis induced by As_2_O_3_ requires negative regulation of both kinases [100]. Finally, the same authors demonstrated that treating BCR-ABL-expressing cells with As_2_O_3_ resulted in paradoxical phosphorylation/activation of mTOR and S6K1 [100], a mechanism that may participate in the reported carcinogenic effects of this pollutant in various settings [100].

Other pollutant categories include pesticides, which can modulate ribosomal protein kinase activity. One of the earliest studies on pesticides and S6K involved rats exposed to cycloheximide, where the authors purified and cloned mitogen/oncogene-activated S6 kinase and identified its active phosphorylation sites [94,101]. Adult zebrafish treated for five days with bifenthrin at various concentrations of 1, 10, and 50 ng/L showed differential gene expression in various genes and related pathways. Among these were the mTOR pathway and all its downstream effectors, including S6K1, the expression of which increased in a dose-dependent manner. Furthermore, these effects persisted for 19 days after bifenthrin withdrawal and recovery. Finally, these alterations were associated with neurobehavioral responses in adult zebrafish [102]. Silkworm *Bombyx mori* exposed to pyriproxyfen at 0.01 µg/L for 96 h had a 5.81-fold higher expression in S6K1 than untreated controls, accompanied by changes in the main immune signaling pathways [103]. Exposure of fall armyworm *Spodoptera frugiperda* to the insecticide chlorantraniliprole for 4 h at 0.024–15 mg/L diet suppressed ribosomal protein S6 kinase 1 (*sfS6K1*) in female adults, followed by downstream vitellogenin *sfvg* downregulation [104]. Bovine mammary epithelial cells (MAC-T) treated with various doses of the herbicide Bifenox (0.2–10 Mg/L) for 48 h experienced impaired cell cycle at sub-G1 and G1 phases, impaired mitochondrial membrane potential, disrupted calcium homeostasis, and hyperphosphorylation of p70 ribosomal S6 kinase [105]. A reduction in phosphorylation of protein kinase B (AKT) and S6K was observed in different cell models of adipocytes (3T3-L1), hepatocytes (HepG2), and myotubes (C2C12) treated with imidacloprid (neonicotinoid insecticide) in a dose-dependent manner at concentrations of 10 and 20 µM. This inhibition was associated with the induction of insulin resistance and a dysregulated insulin signaling pathway [106].

Collectively, different environmental contaminants show different impacts on S6K activity, ranging from affecting gene expression (i.e., PAH and pesticide exposure) to modulating its phosphorylation (i.e., heavy metals). In a real-case scenario of environmental exposure, humans and other organisms can be impacted simultaneously by different pollutants, which can impact S6K activity at various levels, posing a significant potential carcinogenic risk.

**Figure 7:**
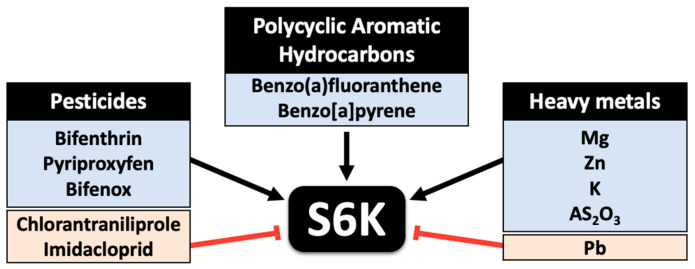
The effects of various environmental pollutants on S6K activity. Polycyclic aromatic hydrocarbons and pesticides impact S6K gene expression, while heavy metals impact S6K phosphorylation. Pesticides such as chlorantraniliprole and imidacloprid inhibit S6K mRNA expression and phosphorylation, respectively. Lead exposure is associated with decreased S6K phosphorylation.

Despite the lack of direct evidence for the impact of different environmental pollutants on carcinogenesis via ribosomal protein kinases, we still postulate its existence for the following reasons: (a) environmental pollutants affect the PI3K and mTOR pathways, autophagy, apoptosis, and cell cycle progression, which are all implicated in carcinogenesis [107,108,109,110], (b) impact, both short- and long-term, ribosomal protein kinases in different in vivo and in vitro models indicating a conserved molecular mechanism, (c) S6Ks promote cell survival and growth in multiple systems and promote cancer progression, (d) S6K1 and S6K2 are isoforms with high structural and functional similarity so that environmental pollutants affecting S6K1 could potentially impact S6K2 as well, (e) as highlighted in our integrative review, S6K2 significantly contributes to carcinogenicity and aggressiveness in various cancers. Importantly, environmental pollutants are known to promote cancer progression [90,111], affect patient responses to chemotherapy [112], and induce severe cancer cases following chronic exposure [113]. Furthermore, environmental pollutants have been shown to impact different oncogene isoforms, such as the estrogen receptor [114] and p53 [115]. Identifying all possible modulators of S6K2 activity, including environmental pollutants, is therefore, crucial to better understand the impact that these may have on health, and may be the starting point for medicinal chemistry projects to obtain drugs that regulate the activity of this kinase. Hence, detailed studies to confirm the direct or indirect association between environment pollutants, S6K, and carcinogenesis should be urgently undertaken. Figure 7 summarizes the effects of various environmental pollutants on S6K activity.

## 6. Homology Modeling and Dynamics Simulations of S6K2 Protein

**Figure 8:**
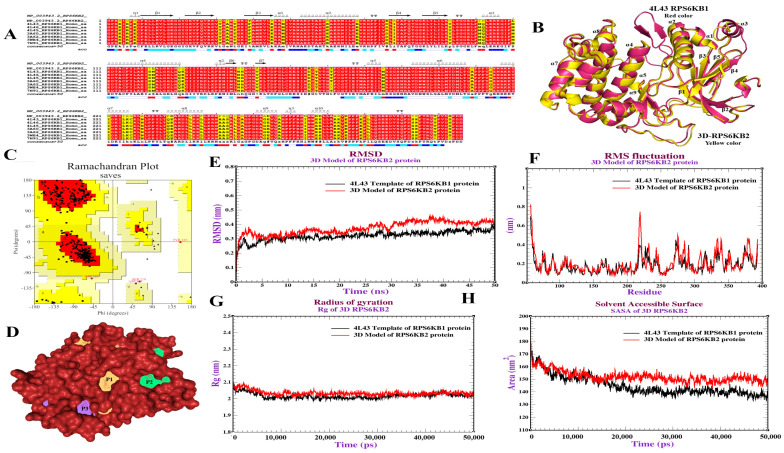
(**A**) Multiple sequence alignment of amino acids of S6K2 protein with structures in the Protein Data Bank. (**B**) Superimposition of a model of S6K2 protein and a template with a highly similar S6K1 (PDB: ID 4L43). (**C**) Ramachandran plot for validation of the 3D model of S6K2 protein using the PROCHECK server. (**D**) Prediction of binding pockets of the target S6K2 protein. (**E**–**H**): Molecular dynamics simulations of S6K2 protein using Gromacs, (**E**): Root Mean Square Deviation (RMSD) of the backbone conformation of a model of S6K2 protein and a highly similar S6K1 (PDB: ID 4L43), (**F**): Root Mean Square Fluctuation (RMSF) of S6K2 protein and a highly similar template S6K1 (PDB: ID 4L43), (**G**): Radius of Gyration (Rg) of both a model of S6K2 protein and a highly similar S6K1 (PDB: ID 4L43), (**H**): Solvent-accessible surface area (SASA) analysis of both S6K2 and S6K1 (PDB: ID 4L43).

Computational approaches, such as molecular modeling, structure-based virtual screening, molecular docking, and molecular dynamics, provide unique insights into the conformational landscape of kinases, structural requirements for inhibitory activity, binding modes, and atomistic mechanisms of allostery, which are critical for rational de novo design [116]. To the best of our knowledge, since the 3D structure of S6K2 is not yet available, the high similarity between the S6K2 and S6K1 domains enables the prediction of S6K2 protein 3D structures using multiple structure alignments. Figure 8A depicts the S6K2 protein with seven β-sheet strands and 10 α-helixes. The predicted 3D structure of the S6K2 protein is illustrated in Figure 8B using the Modeller program. PROCHECK analysis of the Ramachandran plot for this 3D model was performed to investigate its accuracy. It was revealed that 88.5% of the residues fell in the most favored regions, 10.1% in additional allowed regions, 1.0% in generously allowed regions, and 0.3% in disallowed regions, indicating that the generated model was of overall good quality (Figure 8C). Furthermore, the potential binding pockets of S6K2 were identified using the DoGSiteScorer tool (Figure 8D). We determined six predicted pockets and sorted them by druggability score before selecting the highest-scored pocket to perform our docking analysis. Finally, Molecular Dynamics (MD) simulation analysis revealed that the S6K2 structure in aqueous solvation experienced compression during conformational relaxation. Moreover, Figure 8E shows that the RMSD (Root Mean Square Deviation) values for both proteins (S6K2 protein and 4L43 template of S6K1) fluctuate around 2.0 nm after the initial phase, indicating some degree of stability after equilibration. For RMS fluctuations, both proteins show similar fluctuation patterns but with some differences in amplitude, indicating similar but not identical flexibility profiles (Figure 8F). The radius of gyration (Rg) graph suggests that the overall compactness of the two proteins remains relatively constant during the simulation with an Rg value of around 2.0 nm (Figure 8G). Solvent-Accessible Surface Area (SASA) value suggests that the 3D model of S6K2 is more exposed than that of S6K1 (Figure 8H). Overall, MD analysis indicates that the S6K2 3D model exhibits minor structural differences and possibly higher flexibility or less compactness compared to the 4L43 template of S6K1. The similar patterns of flexibility regions for both proteins suggest that these regions may be conserved in their dynamic behavior.

## 7. Structure-Based Design of Ribosomal S6 Kinase Beta 2 (S6K2) Inhibitors

Various computational approaches, such as ensemble-based virtual screening, 3D-QSAR pharmacophore modeling, hybrid virtual screening, docking, and molecular dynamics simulations, have been employed in different studies to discover potential S6K1 inhibitors [1]. The development of new inhibitors for S6K1 could advance the field of targeted cancer therapy [117,118,119]. Unlike S6K1, studies on S6K2 are limited. In 2024, Yu et al. virtually screened 1,575,957 active molecules sourced from the ChemDiv databank. Only four compounds were identified as promising inhibitors of S6K2 [120]. Only two inhibitors, FL772 and PF-4708671, have demonstrated significant selectivity for S6K1 over S6K2 in biochemical assays. No selective inhibitor that specifically targets S6K2 has been identified. The kinase domains of S6K2 and S6K1 exhibit only a few structural differences, posing challenges for the development of small molecules that selectively target either isoform.

### 7.1. Molecular Docking of S6K2 Protein with Active Compounds from the PubChem Database

**Figure 9:**
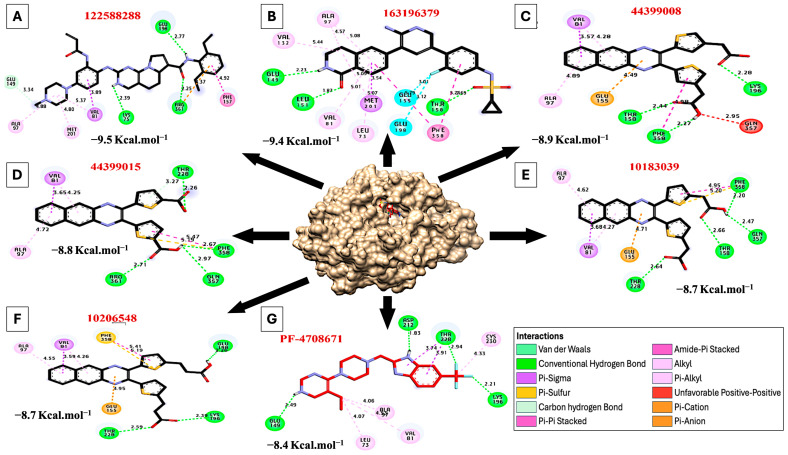
Docking and 3D representations of active compounds (represented in Table 3) from the PubChem database with residues of the S6K2 protein. (**A**) Molecular interactions of amino acids of S6K2 protein with PubChem CID 122588288, (**B**) PubChem CID 163196379, (**C**) PubChem CID 44399008, (**D**) PubChem CID 44399015, (**E**) PubChem CID 10183039, (**F**) PubChem CID 10206548, and (**G**) PF-4708671.

The active compounds targeting S6K1 and S6K2 were selected from the PubChem database. Among these compounds, 12 showed exclusive activity toward S6K2 (Table 1). Molecular docking was utilized to predict the interactions between these compounds and S6K2. This analysis revealed that compounds with PubChem CID 122588288, 163196379, 44399008, 44399015, 10183039, and 10206548 showed favorable predicted binding affinity values of −9.50, −9.40, −8.90, −8.80, −8.70, and −8.70, kcal·mol^−1^ toward S6K2, respectively, compared to PF-4708671 (−8.40 kcal·mol^−1^) (Table 2 and Figure 9). During binding, these compounds formed hydrogen bonds with Glu198, Arg361, Lys75, Leu151, Glu149, Thr158, Phe358, Lys196, Arg361, Thr228, and Glu357. Additionally, hydrophobic interactions were observed, including (Pi-alkyl) interactions with Met201, Ala97, Val81, Val132, Met201, Leu73, Leu148, Lys99, and Leu151; (Sulfur) interactions with Phe358; (Carbon Hydrogen bond) interactions with Glu119, Thr211, Glu149; (Pi-cation) interactions with Arg361 and Glu155; and (Pi-sigma) interactions with Val81 (Table 3). These findings suggest that compounds with CID 122588288 and 163196379 hold particular promise as potential S6K2 inhibitors.

### 7.2. Molecular Docking of S6K2 Protein with Environmental Contaminants

**Figure 10:**
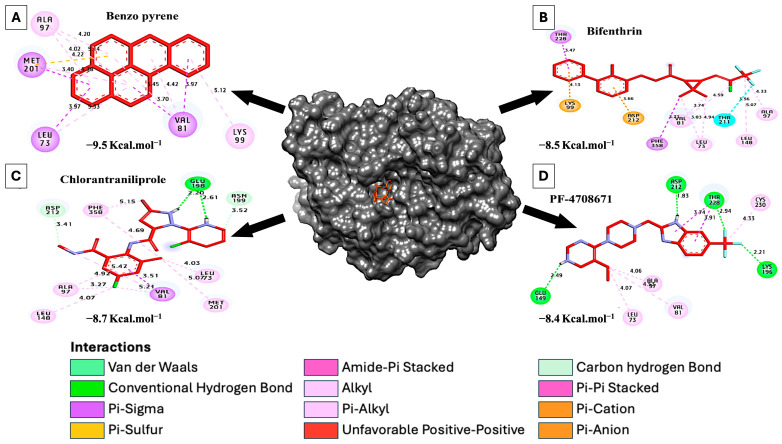
Docking and 3D representations of environmental contaminants (presented in Table 4) with residues of S6K2 protein. (**A**) Molecular interactions of amino acids of S6K2 protein with benzo-a-pyrene, (**B**) bifenthrin, (**C**) chlorantraniliprole, and (**D**) PF-4708671.

Exposure to polyaromatic hydrocarbons (PAHs) can stimulate S6K activity through various mechanisms, including activation of AhR, generation of ROS, and interference with negative regulators of the mTORC1 pathway [121,122]. The results of the docking analysis with selected PAHs showed that benzo-a-pyrene, chlorantraniliprole and bifenthrin molecules presented favorable binding activity with predicted affinity values of −9.50, −8.70, −8.50, and- kcal·mol^−1^ toward S6K2, respectively, compared with PF-4708671 (−8.40 kcal·mol^−1^) (Table 4 and Figure 10). Only chlorantraniliprole formed two hydrogen bonds with Glu198. Additionally, hydrophobic interactions include (Pi-alkyl) with Lys99, Ala97, Leu73, Leu148, Met201, Phe358, and Val81, (Pi-Sigma) with Val81, Met201, Leu73, Phe358, Thr228, (Pi-Cation) with Asp212, (Pi-Anion) with Lys99, (Carbon Hydrogen bond) with Asn199, and Asp212. In comparison, PF-4708671 formed four hydrophilic bonds with Asp212, Thr228, Lys196, and Glu149and six hydrophilic bonds including (Pi-alkyl) with Leu73, Ala97, Val81, Cys230, (Pi-sigma) with Thr228.

## 8. Conclusions

This comprehensive review elucidates the complex regulation of S6K2 through upstream effectors and PTMs, as well as insights gained from in silico analyses, which provide a detailed understanding of S6K2 conformational flexibility and potential binding sites. The structure-based design of S6K2 inhibitors highlights promising avenues for therapeutic development, particularly in targeting diseases such as cancer, in which S6K2 dysregulation has been implicated. Overall, this review presents a holistic overview of S6K2 regulation and function, suggesting this kinase as a pivotal therapeutic target and laying the groundwork for future research and development of targeted therapies.

## 9. Future Perspectives

Although substantial progress has been made in understanding S6K2 biology, significant gaps remain. Key questions persist regarding the distinct roles of S6K2 compared to its closely related isoform S6K1 in cellular signaling and cancer progression. For instance, how specific post-translational modifications such as methylation, phosphorylation, and acetylation impact S6K2 nuclear localization, protein interactions, and functional specificity remains to be fully elucidated. The development of inhibitors represents a major challenge for targeting S6K2. Current inhibitors often lack selectivity between S6K2 and S6K1 due to their structural similarity. Leveraging insights from molecular docking and dynamics simulations, as explored in this review, may help identify novel binding sites or interaction dynamics unique to S6K2. Future efforts should focus on designing highly selective inhibitors through advanced computational modeling and experimental validation, with an emphasis on overcoming the challenges posed by isoform overlap. In addition, a better understanding of S6K2’s role in cancer is critical to drive therapeutic innovation, as this kinase is still underestimated as a clinically relevant drug target. Studies have shown its involvement in regulating chemoresistance, survival pathways, and cell migration, suggesting that it is an attractive target in multiple cancer types. Combining S6K2-targeted therapies with existing treatments, such as mTOR inhibitors or chemotherapeutic agents, holds promise for improving patient outcomes. Another compelling avenue of research is the interaction between S6K2 and environmental factors. Evidence suggests that pollutants, including polycyclic aromatic hydrocarbons and heavy metals, can influence S6K2 activity and potentially contribute to carcinogenesis. Exploring these connections may provide new insights into cancer prevention and therapeutic interventions. By addressing these unanswered questions, future research will further highlight the relevance of S6K2 in homeostasis and physiopathology, ultimately driving the development of innovative treatments for various diseases associated with dysregulated S6K2 activity.

## Figures and Tables

**Table 1:** Compounds exhibiting activity exclusively with S6K2 and not with S6K1 identified from the PubChem database. SwissTarget Prediction: prediction of S6K2 target for the compound using SwissTarget Prediction.

N	ID	Compound	PubChem Reference	Ac_Value ^#^	Ac_Name ^#^	SwissTarget Prediction ^##^	Other S6K SwissTarget Prediction ^##^
1	9950039	2,3-Di-thiophen-2-yl-benzo[g]quinoxaline	bioassay/242021	1.36 µM	IC50 *	0.91	NA
2	10164781	3-(5-(3-[5-(2-Methoxycarbonyl-ethyl)-thiophen-2-yl]-benzo[g]quinoxalin-2-yl)-thiophen-2-yl)-propionic acid methyl ester	bioassay/242021	6.13 µM	IC50 *	0.72	NA
3	10183039	(5-[3-(5-Carboxymethyl-thiophen-2-yl)-benzo[g]quinoxalin-2-yl]-thiophen-2-yl)-acetic acid	bioassay/242021	1.28 µM	IC50 *	1.0	NA
4	10206548	3-(5-(3-[5-(2-Carboxy-ethyl)-thiophen-2-yl]-benzo[g]quinoxalin-2-yl)-thiophen-2-yl)-propionic acid	bioassay/242021	0.87 µM	IC50 *	0.81	NA
5	44399002	6,7-Di-thiophen-2-yl-2,3-dihydro-1,4-dioxa-5,8-diaza-anthracene-2-carboxylic acid	bioassay/242021	0.95 µM	IC50 *	1.0	NA
6	44399008	(5-[3-(4-Carboxymethyl-thiophen-2-yl)-benzo[g]quinoxalin-2-yl]-thiophen-3-yl)-acetic acid	bioassay/242021	0.64 µM	IC50 *	1.0	NA
7	44399015	5-[3-(5-Carboxythien-2-yl)benzo[g]quinoxalin-2-yl]thiophene-2-carboxylic acid	bioassay/242021	10 µM	IC50 *	0.1	NA
8	44399109	4-(5-(3-[5-(3-Carboxy-propyl)-thiophen-2-yl]-benzo[g]quinoxalin-2-yl)-thiophen-2-yl)-butyric acid	bioassay/242021	0.82 µM	IC50 *	0.91	NA
9	44399200	7,8-Dimethoxy-2,3-di-thiophen-2-yl-pyrazino [2,3-b]quinoxaline	bioassay/242021	1.14 µM	IC50 *	1.0	RPS6KA2; 0.12
10	135964360	2,3-Di-thiophen-2-yl-quinoxaline-6,7-diol	bioassay/242021	9.61 µM	IC50 *	1.0	RPS6KA3; 0.11
11	122588288	N-(4-(6-amino-5-(1-oxo-1,2,3,4-tetrahydroisoquinolin-6-yl)pyridin-3-yl)-3-fluorophenyl) cyclopropanesulfonamide	bioassay/1746035	5.5 µM	%Inhibition **	N/A	RPS6KB1; 0.1
12	163196379	N-(2,6-diethylphenyl)-2-[4-(4-methylpiperazin-1-yl)-2-(prop-2-enoylamino)anilino]-5,6-dihydropyrimido [4,5-e]indolizine-7-carboxamide	bioassay/1819637	1.65 µM	IC50 ***	N/A	RPS6KA1, RPS6KA2, RPS6KA4, RPS6KA5, RPS6KA6; 0.06

* Assay description: In vitro inhibition of SR protein kinase 1 by compound dissolved in 100% DMSO using 5″-[gamma-33P]-triphosphate; ** Assay description: Inhibition of p70s6kb in human NCI-H929 cells by mass spectroscopic analysis; *** Assay description: Inhibition of human recombinant P70S6Kbeta expressed in Sf9 insect cells using [KKRNRTLTK] as substrate in the presence of [33P]-ATP by radiometric hotspot kinetic assay; ^#^ Ac_value (Activity Value): This field contains the “numerical result” of the measured activity. It corresponds to the value associated with the “Ac_name”. Ac_name (Activity Name): This field specifies the “type of activity” being measured in the assay; ^##^ SwissTarget Prediction: Probability of the query molecule to have this protein as a target. A probability of 0.9 for a specific target indicates that there is a 90% likelihood that the compound interacts with that target. This prediction is based on the similarity of the compound’s structure to known bioactive compounds in the database, as well as machine learning algorithms.

**Table 2:** Binding affinity of S6K2 protein with active compounds from the PubChem database.

PubChem CID	Ligand Name	Binding Affinity
122588288	N-(4-(6-amino-5-(1-oxo-1-2-3, 4-tetrahydroisoquinoline-6-yl) pyridine-3-yl)-3-fluorophenyl) cyclopropane sulfonamide	−9.5
163196379	N-(2,6-diethyl phenyl)-2- [4-(4-methyl piperazine-1-yl)-2-(prop-2-enoylamino) anilino]-5-6-dihydropyrimido [45-e] indolizine-7-carboxamide	−9.4
44399008	5-[3-(4-Carboxymethyl-thiophene-2-yl)-benzo[g]quinoxalin-2-yl]-thiophen-3-yl)-acetic_acid	−8.9
44399015	5-[3-(5-Carboxythien-2-yl) benzo[g]quinoxalin-2-yl] thiophene-2-carboxylic_acid	−8.8
10183039	5-[3-(5-Carboxymethyl-thiophene-2-yl)-benzo[g]quinoxalin-2-yl]-thiophen-2-yl)-acetic_acid	−8.7
10206548	3-(5-(3-[5-(2-Carboxy-ethyl)-thiophen-2-yl]-benzo[g]quinoxalin-2-yl)-thiophene-2-yl)-propionic_acid	−8.7

**Table 3:** Interactions of S6K2 protein with active compounds from the PubChem database.

	PubChem CID	3D Structure	Hydrophilic Interactions		Hydrophobic Contacts		No. of H-Bonds	No. of Total Bonds	Affinity kcal mol^−1^
			Residue (H-Bond)	Length	Residue (Bond Type)	Length			
1	122588288	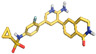	Glu198, (H-Bond) Arg361, (H-Bond) Lys75, (H-Bond)	2.77 2.25 2.39	Met201, (Pi-alkyl) Ala97, (Pi-alkyl) Val81, (Pi-alkyl) Val81, (Pi-Sigma) Glu149, (Carbon H-bond) Arg361, (Pi-cation) Phe157, (Pi stacked bond)	4.80 4.88 5.37 3.89 3.34 4.37 4.92	3	10	−9.50
2	163196379	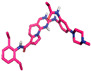	Thr158, (H-Bond) Leu151, (H-Bond) Glu149, (H-Bond)	4.69 1.87 2.23	Val132, (Pi-alkyl) Ala97, (Pi-alkyl) Met201, (Pi-alkyl) Leu73, (Pi-alkyl) Val81, (Pi-alkyl) Met201, (Pi-Sigma) Phe358, (Pi stacked bond) Glu155, (Halogen) Glu198, (Halogen)	5.44 4.57 5.09 5.07 5.01 3.54 5.22 3.12 3.01	3	12	−9.40
3	44399008	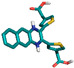	Thr158, (H-Bond) Phe358, (H-Bond) Lys196, (H-Bond)	2.44 2.27 2.28	Ala97, (Pi-alkyl) Val81, (Pi-alkyl) Val81, (Pi-sigma) Glu155, (Pi-Cation) Phe358, (Pi stacked bond) Gln357, (Unfavorable bond)	4.89 4.28 3.57 4.49 4.98 2.95	3	9	−8.90
4	44399015	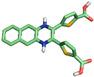	Arg361, (H-Bond) Thr228, (H-Bond) Phe358, (H-Bond) Glu357, (H-Bond)	2.71 2.26 2.67 2.97	Ala97, (Pi-alkyl) Val81, (Pi-alkyl) Val81, (Pi-sigma) Phe358, (Sulfur) Phe358, (Pi stacked bond)	4.72 4.25 3.65 5.19 5.47	4	9	−8.80
5	10183039	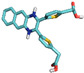	Thr158, (H-Bond) Thr228, (H-Bond) Phe358, (H-Bond) Glu357, (H-Bond)	2.66 2.64 2.20 2.47	Ala97, (Pi-alkyl) Val81, (Pi-alkyl) Val81, (Pi-sigma) Phe358, (Sulfur) Phe358, (Pi stacked bond) Glu155, (Pi-Cation)	4.62 4.27 3.68 5.20 4.95 4.71	4	10	−8.70
6	10206548	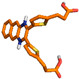	Thr228, (H-Bond) Lys196, (H-Bond) Glu198, (H-Bond)	2.59 2.39 2.26	Ala97, (Pi-alkyl) Val81, (Pi-alkyl) Val81, (Pi-sigma) Phe358, (Sulfur) Phe358, (Pi stacked bond) Glu155, (Pi-Cation)	4.55 4.26 3.59 5.19 5.41 4.95	3	9	−8.70
7	PF-4708671	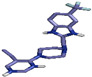	Asp212, (H-Bond) Thr228, (H-Bond) Lys196, (H-Bond) Glu149, (H-Bond)	1.83 2.94 2.21 2.49	Leu73, (Pi-alkyl) Ala97, (Pi-alkyl) Val81, (Pi-alkyl) Cys230, (Pi-alkyl) Thr228, (Pi-sigma) Thr228, (Pi-sigma)	4.07 4.06 4.53 4.33 3.74 3.91	4	10	−8.40

**Table 4:** Interactions of environmental contaminants compounds with residues of S6K2 protein.

	Ligand (PubChem CID)	3D Structure	Hydrophilic Interactions		Hydrophobic Contacts		No. of H-Bonds	No. of Total Bonds	Affinity kcal mol^−1^
			Residue (H-Bond)	Length	Residue (Bond Type)	Length			
1	Benzo-a-pyrene (2336)	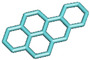	-	-	Lys99, (Pi-alkyl) Ala97, (Pi-alkyl) Ala97, (Pi-alkyl) Met201, (Pi-Sigma) Leu73, (Pi-Sigma) Val81, (Pi-Sigma) Val81, (Pi-Sigma) Ala97, (Pi-alkyl) Met201, (Pi-alkyl) Met201, (Pi-cation) Leu73, (Pi-alkyl) Val81, (Pi-alkyl) Val81, (Pi-alkyl)	5.12 4.20 4.02 3.40 3.97 3.70 3.97 4.22 3.30 4.22 5.35 5.45 4.42	0	13	−9.50
2	Chlorantraniliprole (11271640)	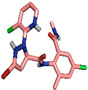	Glu198, (H-Bond) Glu198, (H-Bond)	2.61 2.20	Leu73, (Pi-alkyl) Leu148, (Pi-alkyl) Ala97, (Pi-alkyl) Met201, (Pi-alkyl) Phe358, (Pi-alkyl) Val81, (Pi-alkyl) Val81, (Pi-Sigma) Asn199, (Carbon H-Bond) Asp212, (Carbon H-Bond) Phe358, (Pi-alkyl) Ala97, (Pi-alkyl) Val81, (Pi-alkyl)	4.03 4.07 3.27 5.00 5.15 5.21 3.51 3.52 3.41 4.69 4.96 3.51	2	14	−8.70
3	Bifenthrin (6442842)	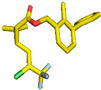	-	-	Leu73, (Pi-alkyl) Leu148, (Pi-alkyl) Ala97, (Pi-alkyl) Val81, (Pi-alkyl) Phe358, (Pi-sigma) Thr228, (Pi-sigma) Lys99, (Pi-Anion) Asp212, (Pi-Cation) Thr211, (Halogen) Leu73, (Pi-alkyl) Val81, (Pi-alkyl)	4.94 5.07 4.33 3.74 3.73 3.47 4.13 3.66 3.56 3.03 4.59	0	11	−8.50
4	PF-4708671 (51371303)	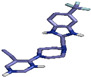	Asp212, (H-Bond) Thr228, (H-Bond) Lys196, (H-Bond) Glu149, (H-Bond)	1.83 2.94 2.21 2.49	Leu73, (Pi-alkyl) Ala97, (Pi-alkyl) Val81, (Pi-alkyl) Cys230, (Pi-alkyl) Thr228, (Pi-sigma) Thr228, (Pi-sigma)	4.07 4.06 4.53 4.33 3.74 3.91	4	10	−8.40

## Data Availability

The data presented in the study are included in the article/Supplementary Material, and further inquiries can be directed to the corresponding authors.

## Supplementary Materials

The following supporting information can be downloaded at https://www.mdpi.com/article/10.3390/ijms26010176/s1.
